# Usefulness of retroperitoneoscopic renal needle biopsy for patients with contraindications for percutaneous renal biopsy

**DOI:** 10.1007/s11255-019-02303-1

**Published:** 2019-10-03

**Authors:** Toshihiro Shimizu, Yoshitaka Kinoshita, Taro Kubo, Takahiro Shinzato, Koji Nanmoku, Takashi Yagisawa

**Affiliations:** Surgical Branch, Institute of Kidney Diseases, Jichi Medical University Hospital, Yakushiji 3311-1, Shimotsuke, Tochigi 329-0498 Japan

Editor,

Renal biopsy is essential for the diagnosis of renal diseases, and percutaneous renal biopsy (PRB) is currently the standard procedure [1]. However, some patients have absolute or relative contraindications to PRB [2]. We performed retroperitoneoscopic renal needle biopsy (RPNB) for such patients. Here, we present our technique for RPNB and discuss its outcomes.

**Fig. 1:**
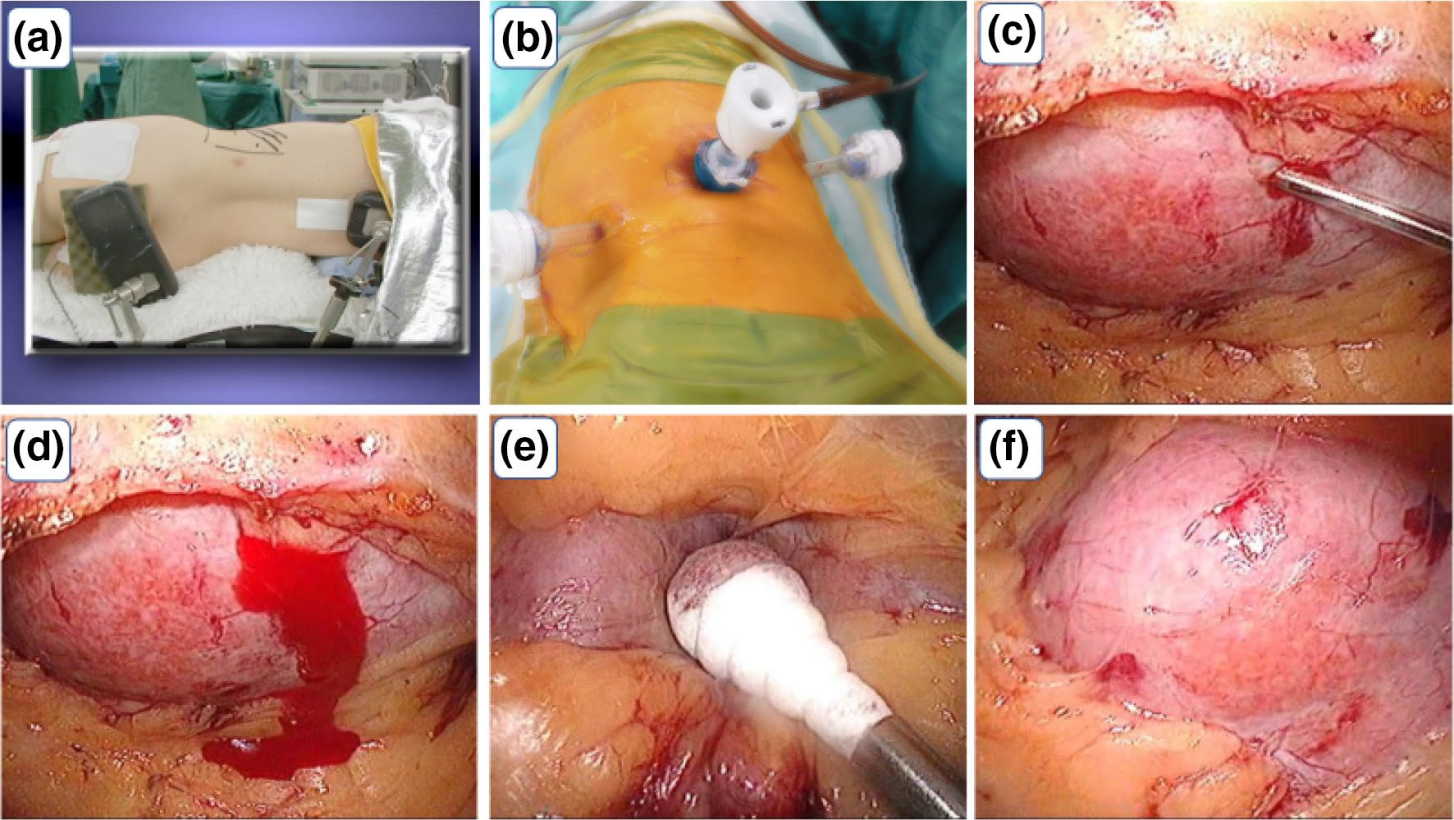
Operative technique. **a** Full lateral position. **b** 2- or 3-port set. **c** The biopsy needle (18 G) is advanced to the kidney through the port, and inserted. **d** A small amount of bleeding from the insertion point. **e** The insertion point is pressed for approximately 10 min. **f** Haemostasis is obtained; we obtained two or three biopsies

From 2014 to 2018, we performed RPNB on 47 patients with contraindications for PRB. The kidney was approached via a laparoscopic retroperitoneal route with the patients placed in the flank position under general anaesthesia (Fig. 1a, b). The first port (ϕ10 mm) was placed at the lower edge of the 11th rib, and the second port (ϕ12 mm) was placed between the 12th rib and the iliopsoas muscle. If surgical operation with two ports was difficult, we placed a third port (ϕ5 mm or ϕ12 mm) at the abdomen about 8 cm from the first port. Two or three needle biopsies were obtained from the lower pole of the kidney through the second port. Haemostasis was achieved by applying pressure to the insertion point of the needle under direct vision for approximately 10 min (Fig. 1c–f). There was no need to retain the drain, as we confirmed haemostasis. The patients were on bed rest after surgery until the next morning, taking care to not compress the flank.

The most common reason for choosing RPNB was obesity in 15 patients (31.9%), and the mean BMI of obese patients was 32.7 ± 4.6 kg/m^2^ (range 26.1–43.7 kg/m^2^). Other reasons included coagulation abnormalities in 10 patients (21.2%), multiple kidney cysts in 9 patients (19.1%), solitary kidney in 6 patients (12.7%), and difficulty holding body position during renal biopsy under local anaesthesia in 4 patients. Additionally, one patient could not hold his breath temporarily during renal biopsy, the kidneys of one patient already exhibited atrophy, and one patient had aberrant anatomy with the intestinal tract near the kidneys.

The median number of glomeruli collected was 29 (range 2–87), which was sufficient for diagnosis. One obese patient lost approximately 50 ml of blood, and we applied pressure to the puncture site for an additional 15 min to achieve haemostasis. Notably, there were no complications that were directly related to the procedure.

Previous studies have indicated that PRB is a relatively safe procedure with a low risk of complications [1, 2]. However, some patients have absolute or relative contraindications to PRB, and the frequency of bleeding complications requiring blood transfusion after PRB was reported to be 0.2–4.7% [1, 3–6]. The advantage of RPNB is that it is possible to avoid the risk of accidentally damaging the main blood vessels and to confirm haemostasis by looking directly at the puncture site. In conclusion, RPNB is a useful alternative method for patients who have difficulty undergoing PRB for various reasons.
